# Features of Invariant Extended Kalman Filter Applied to Unmanned Aerial Vehicle Navigation

**DOI:** 10.3390/s18092855

**Published:** 2018-08-29

**Authors:** Nak Yong Ko, Wonkeun Youn, In Ho Choi, Gyeongsub Song, Tae Sik Kim

**Affiliations:** 1 Department of Electronic Engineering, Chosun University, 375 Seosuk-dong Dong-gu, Gwangju 501-759, Korea; 2 Korea Aerospace Research Institute, Daejon 34133, Korea; wkyoun@kari.re.kr (W.Y.); inho@kari.re.kr (I.H.C.); kts@kari.re.kr (T.S.K.); 3 Department of Control and Instrumentation Engineering, Chosun University, 375 Seosuk-dong Dong-gu, Gwangju 501-759, Korea; sgs0369@naver.com

**Keywords:** invariant extended Kalman filter, unmanned aerial vehicle, location, attitude, velocity, GPS, MEMS-AHRS, Kalman gain, error covariance, innovation, estimation

## Abstract

This research used an invariant extended Kalman filter (IEKF) for the navigation of an unmanned aerial vehicle (UAV), and compared the properties and performance of this IEKF with those of an open-source navigation method based on an extended Kalman filter (EKF). The IEKF is a fairly new variant of the EKF, and its properties have been verified theoretically and through simulations and experiments. This study investigated its performance using a practical implementation and examined its distinctive features compared to the previous EKF-based approach. The test used two different types of UAVs: rotary wing and fixed wing. The method uses sensor measurements of the location and velocity from a GPS receiver; the acceleration, angular rate, and magnetic field from a microelectromechanical system-attitude heading reference system (MEMS-AHRS); and the altitude from a barometric sensor. Through flight tests, the estimated state variables and internal parameters such as the Kalman gain, state error covariance, and measurement innovation for the IEKF method and EKF-based method were compared. The estimated states and internal parameters showed that the IEKF method was more stable and convergent than the EKF-based method, although the estimated locations, velocities, and altitudes of the two methods were comparable.

## 1. Introduction

The invariant extended Kalman filter is a fairly recent development that improves the extended Kalman filter using the geometrically transformed state error and output error based on the Lie group theory [1]. It could be regarded as an extension and generalization of the multiplicative extended Kalman filter [2,3,4,5]. It can be derived and applied using either the abstract Lie group [6] or matrix Lie group formulation [7]. Derivations of the invariant extended Kalman filter (IEKF) for use in the navigation of an unmanned aerial vehicle using measurements of the location and velocity from a GPS; the angular rate, acceleration, and magnetic field from a microelectromechanical system-attitude heading reference system (MEMS-AHRS); and the altitude from a barometric sensor have been reported [2,8]. These were based on the abstract Lie group approach. A matrix Lie group formulation of the IEKF was used for simultaneous localization and mapping (SLAM) and improved the consistency of the estimation compared to the EKF-based SLAM method [9].

The IEKF approach has several distinctive advantageous features compared to the usual EKF-based approaches: (1) a symmetry preserving observer, (2) sound geometric structures for quaternion estimation, and (3) a large expected domain of convergence [8]. The IEKF-based method preserves the norm of the estimated quaternion as a unit. In contrast, the prediction and correction procedures of the usual EKF break the unit norm constraint on the quaternion for the attitude. In addition, the approximation to a linear system of a nonlinear process and measurement model sometimes induces an unstable and divergent estimation. The IEKF relieves this problem by extending the state space where convergence is preserved.

The IEKF is basically an invariant observer which adopts an EKF approach for determining observer gain to take advantage of the convergence property of the *linear* Kalman filter. The property of *invariant,* or otherwise called *symmetry-preserving* system had been used for control applications [10]. Aghannan and Rouchon [11] initially exploited the invariance property for observer or estimator design resulting in an invariant observer. Though the invariant observer proposed in [11] was the predecessor of the IEKF, it did not incorporate the idea of EKF. The invariant observer was further developed and convergence property was verified by numerical simulation using a mechanical system of ball and beam [12]. Martin et al. introduced *invariant output error* in their development of an invariant tracking method [13]. Though the invariant output error was proposed and used for control use, it played an important role in development of IEKF later [14]. The invariant observer was theoretically further investigated, providing the foundations for development into IEKF, especially for navigation applications [15]. Finally, IEKF was proposed by incorporating the EKF methodology into the invariant observer for determining the observer gain [14]. It should be noticed that the IEKF does not use direct linearization of nonlinear process model and measurement model, unlike the usual EKF [16]. This will be discussed in Section 3.1 along with the explanation about the linearization process.

Studies on the IEKF have demonstrated the feasibility of using it for aerial, indoor, and road navigation [17]. The IEKF and EKF were applied for SLAM, and a comparison of their results proved that the IEKF improved the estimation convergence and consistency compared to the EKF [18]. The comparison used simulated data. The IEKF was also used for helicopter unmanned aerial vehicle (UAV) navigation [19], and its estimation performance was found based on experimental results, focusing on the estimated data of the state variables, excluding internal parameters such as the Kalman gain, state error covariance, and measurement innovation. The IEKF was also compared with the multiplicative extended Kalman filter (MEKF) [2,8,20]. Barczyk et al. compared the IEKF with the MEKF for indoor localization using scan matching point clouds captured by a low-cost Kinect depth camera [20]. They used experimental results and compared the Kalman gain for the case of indoor navigation. Barczy and Lynch applied the IEKF to a magnetometer-plus-GPS-aided inertial navigation system for a helicopter UAV, and presented experimental data on the estimated state variables [21].

This study concentrated on supporting the availability of IEKF navigation by a comparison and analysis of the estimation results from flight tests of UAVs, which are widely used. Experiments using two UAVs, a fixed wing UAV and a rotary wing UAV, compared the features of the IEKF and conventional EKF. In particular, this study compares the internal parameters of the Kalman gain, state error covariance, and measurement innovation to explicitly show the features of the IEKF. Theoretical derivations and simulations have verified the features of the IEKF in numerous studies [1,6,7,8]. However, studies investigating and analyzing the estimation results and internal parameters through practical applications were not sufficient to validate the feasibility of using the IEKF in a wide range of applications. In studies where experimental results were compared, they usually concentrated on the estimated results of state variables, excluding internal parameters such as the Kalman gain, state error covariance, and measurement innovation [19].

Section 2 lists the notations used to describe the IEKF-based navigation methods. Section 3 briefly describes the IEKF-based navigation method used in this research, and also explains the EKF-based method that was used for comparison. Section 4 provides details of the experiments using a fixed wing UAV and quadrotor to verify and compare the methods. Section 5 compares and discusses the properties revealed from the experiments. This section compares the estimation results for the state variables and internal parameters such as the Kalman gain, state error covariance, and measurement innovation. Finally, Section 6 provides some concluding remarks and possible future research directions related to the current research.

## 2. Nomenclature

The following nomenclature is used in the paper.



q(t)

attitude represented by quaternion at time *t*: $q\left(t\right)={\left(q_{0}\left(t\right),q_{x}\left(t\right),q_{y}\left(t\right),q_{z}\left(t\right)\right)}^{T}$
where $q_{0}\left(t\right)$ is the scalar part and $\left(q_{x}\left(t\right),q_{y}\left(t\right),q_{z}\left(t\right)\right)$ is the vector part of the quaternion

x(t)

location at time *t*: $x\left(t\right)={\left(x(t),y(t),z(t)\right)}^{T}$

v(t)

velocity at time *t*: $v\left(t\right)=\frac{dx(t)}{dt}={\left(v_{x}\left(t\right),v_{y}\left(t\right),v_{z}\left(t\right)\right)}^{T}$

ω(t)

rotational velocity measured by gyroscope in the instrument coordinate frame at time *t*:


$\omega \left(t\right)={\left(\omega_{x}\left(t\right),\omega_{y}\left(t\right),\omega_{z}\left(t\right)\right)}^{T}$



a(t)

acceleration measured in the instrument coordinate frame at time *t*:


$a\left(t\right)={\left(a_{x}\left(t\right),a_{y}\left(t\right),a_{z}\left(t\right)\right)}^{T}$



$z_{x}\left(t\right)$

measurement of location at time *t*: $z_{x}\left(t\right)={\left(z_{x}\left(t\right),z_{y}\left(t\right),z_{z}\left(t\right)\right)}^{T}$

$z_{v}\left(t\right)$

measurement of velocity at time *t*: $z_{v}\left(t\right)={\left(z_{v_{x}}\left(t\right),z_{v_{y}}\left(t\right),z_{v_{z}}\left(t\right)\right)}^{T}$

$z_{m}\left(t\right)$

measurement of magnetic field in the instrument coordinate frame at time *t*:


$z_{m}\left(t\right)={\left(z_{m_{x}}\left(t\right),z_{m_{y}}\left(t\right),z_{m_{z}}\left(t\right)\right)}^{T}$



$z_{h}\left(t\right)$

measurement of altitude at time *t*

$b_{h}\left(t\right)$

bias in altitude measurement at time *t*: $z_{h}\left(t\right)=z\left(t\right)+b_{h}\left(t\right)$

$b_{\omega }\left(t\right)$

bias in angular rate measurement at time *t*: $b_{\omega }\left(t\right)={\left(b_{\omega_{x}}\left(t\right),b_{\omega_{y}}\left(t\right),b_{\omega_{z}}\left(t\right)\right)}^{T}$

$s_{a}\left(t\right)$

scale factor in acceleration measurement at time *t*

g

gravitational acceleration regarded as constant throughout the implementation
in the northeast down (NED) coordinate system

m

geomagnetic field appropriate for a location in the NED coordinate frame that is considered
to be constant throughout the experiment: $m={\left(m_{x},m_{y},m_{z}\right)}^{T}$

$e_{d}$

3×1 unit vector in the direction *d*, where d∈{x,y,z}

In the following description, time index (t) will be omitted from the notations if there arises no ambiguity without index (t) in the context. Since the AHRS measures acceleration and angular rate as well as the magnetic field, it is also considered that AHRS includes inertial measurement unit (IMU). Thus, in this paper, IMU measurement means the acceleration and angular rate measured by AHRS.

## 3. IEKF and ecl-EKF for UAV Navigation

This section briefly describes the IEKF-based navigation method adopted in this research and the EKF-based method that was used for comparison. Two IEKF methods were used: one for estimating the location, attitude, and velocity [2], and the other for estimating the attitude and velocity [8]. Both of them specifically used right IEKF methods and were from [2,8], respectively. The method to be compared with the IEKF methods was the EKF from the estimation and control library (ECL) of the PX4 project [22], which will be called the ecl-EKF from now on. Hereafter, the IEKF used for estimating the location, attitude, and velocity will be called the IEKF-lav, while the IEKF for the attitude and velocity will be called the IEKF-av.

### 3.1. IEKF-lav: Right IEKF for Estimation of Location, Attitude, and Velocity

The right IEKF used for estimating the attitude, velocity, and location is first described [2]. The state vector χ to be estimated consists of the state variables shown in Equation (1):

(1) $$\chi ={\left(q,v,x,b_{\omega },s_{a},b_{h}\right)}^{T}.$$

The state is subject to the process model shown as a differential Equation (2):

(2) $$\dot{\chi }=\left(\begin{array}{c} \dot{q}\\ \dot{v}\\ \dot{x}\\ {\dot{b}}_{\omega }\\ {\dot{s}}_{a}\\ {\dot{b}}_{h} \end{array}\right)=\left(\begin{array}{c} \frac{1}{2}q*\left(\omega -b_{\omega }\right)\\ g+\frac{1}{s_{a}}q*a*q^{-1}\\ v\\ 0\\ 0\\ 0 \end{array}\right).$$

The measurements include the velocity and location from the GPS, altitude from a barometric sensor, and magnetic field from a magnetometer in the MEMS-AHRS as follows: $z={\left(z_{v},z_{x},z_{h},z_{m}\right)}^{T}$. The measurements are related to the state as shown by the measurement model (3):

(3) $$z=\left(\begin{array}{c} z_{v}\\ z_{x}\\ z_{h}\\ z_{m} \end{array}\right)=\left(\begin{array}{c} v\\ x\\ x\cdot e_{z}+b_{h}\\ q^{-1}*m*q \end{array}\right).$$

In the application of the right IEKF, the process and measurement model incorporates process noise $w={\left(w_{q},w_{v},w_{x},w_{b_{\omega }},w_{s_{a}},w_{b_{h}}\right)}^{T}$ and measurement noise $\nu ={\left(\nu_{v},\nu_{x},\nu_{h},\nu_{m}\right)}^{T}$ in models (2) and (3), resulting in Equations (4) and (5):

(4) $$\dot{\chi }=\left(\begin{array}{c} \dot{q}\\ \dot{v}\\ \dot{x}\\ {\dot{b}}_{\omega }\\ {\dot{s}}_{a}\\ {\dot{b}}_{h} \end{array}\right)=\left(\begin{array}{c} \frac{1}{2}q*\left(\omega -b_{\omega }\right)\\ g+\frac{1}{s_{a}}q*a*q^{-1}\\ v\\ 0\\ 0\\ 0 \end{array}\right)+\left(\begin{array}{c} M_{q}w_{q}*q\\ M_{v}w_{v}\\ M_{x}w_{x}\\ q^{-1}*M_{b_{\omega }}w_{b_{\omega }}*q\\ s_{a}M_{s_{a}}w_{s_{a}}\\ M_{b_{h}}w_{b_{h}} \end{array}\right)$$

(5) $$z=\left(\begin{array}{c} z_{v}\\ z_{x}\\ z_{h}\\ z_{m} \end{array}\right)=\left(\begin{array}{c} v+N_{v}\nu_{v}\\ x+N_{x}\nu_{x}\\ x\cdot e_{z}+b_{h}+N_{h}\nu_{h}\\ q^{-1}*\left(m+N_{m}\nu_{m}\right)*q \end{array}\right).$$

The inclusion of noise in the process and measurement model, as shown in Equations (4) and (5), transforms the problem for the application of the IEKF, and the IEKF estimates the state and error covariance P as shown in Equations (6) and (7), respectively:

(6) $$\dot{\hat{\chi }}=\left(\begin{array}{c} \dot{\hat{q}}\\ \dot{\hat{v}}\\ \dot{\hat{x}}\\ {\dot{\hat{b}}}_{\omega }\\ {\dot{\hat{s}}}_{a}\\ {\dot{\hat{b}}}_{h} \end{array}\right)=\left(\begin{array}{c} \frac{1}{2}\hat{q}*\left(\omega -{\hat{b}}_{\omega }\right)\\ g+\frac{1}{{\hat{s}}_{a}}\hat{q}*a*{\hat{q}}^{-1}\\ \hat{v}\\ 0\\ 0\\ 0 \end{array}\right)+\left(\begin{array}{c} K_{q}E*\hat{q}\\ K_{v}E\\ K_{x}E\\ {\hat{q}}^{-1}*K_{b_{\omega }}E*\hat{q}\\ {\hat{s}}_{a}K_{s_{a}}E\\ K_{b_{h}}E \end{array}\right),$$

(7) $$\dot{P}=AP+PA^{T}+MM^{T}-PC^{T}{\left(NN^{T}\right)}^{-1}CP,$$

where process error covariance M and measurement error covariance N are given in Equation (8):

(8) $$\begin{array}{c} M=Diag(M_{q},M_{v},M_{x},M_{b_{\omega }},M_{s_{a}},M_{b_{h}}),\\ N=Diag(N_{v},N_{x},N_{h},N_{m}). \end{array}$$

In Equations (6) and (7) for the state χ and error covariance P, the Kalman gain K and output error E are given by Equations (9) and (10), respectively:

(9) $$K=-{\left(K_{q},K_{v},K_{x},K_{b_{\omega }},K_{s_{a}},K_{b_{h}}\right)}^{T}=PC^{T}{\left(NN^{T}\right)}^{-1},$$

(10) $$E=\left(\begin{array}{c} {\hat{z}}_{v}-z_{v}\\ {\hat{z}}_{x}-z_{x}\\ {\hat{z}}_{h}-z_{h}\\ m-\hat{q}*z_{m}*{\hat{q}}^{-1} \end{array}\right)=\left(\begin{array}{c} \hat{v}-v-N_{v}\nu_{v}\\ \hat{x}-x-N_{x}\nu_{x}\\ \left(\hat{x}-x\right)\cdot e_{z}+{\hat{b}}_{h}-b_{h}-N_{h}\nu_{h}\\ m-\hat{q}*q^{-1}*\left(m+N_{m}\nu_{m}\right)*q*{\hat{q}}^{-1} \end{array}\right).$$

The calculation of Kalman gain K and state error covariance P requires linearized state transition coefficient A and measurement coefficient C as shown in Equations (7) and (9). Usual EKF takes the partial derivatives of process model and measurement model with respect to state as the matrices A and C, respectively. On the contrary, IEKF finds the matrices A and C in a different way from the usual EKF because the IEKF takes state error differently from that of the usual EKF [8,16]. To make the observer invariant and adapt the problem for IEKF application, the state error $\eta ={\left(\eta_{q},\eta_{v},\eta_{x},\eta_{b_{\omega }},\eta_{s_{a}},\eta_{b_{h}}\right)}^{T}$ is taken as Equation (11) [2]:

(11) $$\left(\begin{array}{c} \eta_{q}\\ \eta_{v}\\ \eta_{x}\\ \eta_{b_{\omega }}\\ \eta_{s_{a}}\\ \eta_{b_{h}} \end{array}\right)=\left(\begin{array}{c} \hat{q}*q^{-1}\\ \hat{v}-v\\ \hat{x}-x\\ q*\left({\hat{b}}_{\omega }-b_{\omega }\right)*q^{-1}\\ \frac{{\hat{s}}_{a}}{s_{a}}\\ {\hat{b}}_{h}-b_{h} \end{array}\right).$$

Unlike the usual EKF where state error is taken as the algebraic difference between estimated state and true state, the state error in IEKF has a different form as shown at the first, fourth, and fifth components of the state error vector in Equation (11).

For usual EKF, the system equation for state error differential, which is derived from the state estimation equation for usual EKF and the usual process model, is shown as Equation (12) [8]:

(12) $$\delta \dot{\eta }=\left(A-KC\right)\delta \eta -Mw+KN\nu .$$

Likewise, for the IEKF, the system equation for state error differential $\dot{\eta }$ can be found by combining Equations (4), (6), (10), and (11). Linearizing the system equation for state error differential $\dot{\eta }$ of the IEKF and dropping all the quadratic terms for the noise and infinitesimal state error results in a linearized state error differential system, which is equivalent to Equation (12). Finding the correspondence between the linearized state error differential system for IEKF and Equation (12) for usual EKF yields the linearized process transition coefficient A and measurement coefficient C for IEKF as Equations (13) and (14), respectively [2]:

(13) $$A=\left(\begin{array}{cccccc} 0_{33} & 0_{33} & 0_{33} & -\frac{1}{2}I_{3} & 0_{31} & 0_{31}\\ -2{\hat{I}}_{a\times } & 0_{33} & 0_{33} & 0_{33} & -{\hat{I}}_{a} & 0_{31}\\ 0_{33} & I_{3} & 0_{33} & 0_{33} & 0_{31} & 0_{31}\\ 0_{33} & 0_{33} & 0_{33} & {\hat{I}}_{\omega \times } & 0_{31} & 0_{31}\\ 0_{13} & 0_{13} & 0_{13} & 0_{13} & 0_{11} & 0_{11}\\ 0_{13} & 0_{13} & 0_{13} & 0_{13} & 0_{11} & 0_{11} \end{array}\right),$$

(14) $$C=\left(\begin{array}{cccccc} 0_{33} & I_{3} & 0_{33} & 0_{33} & 0_{31} & 0_{31}\\ 0_{33} & 0_{33} & I_{3} & 0_{33} & 0_{31} & 0_{31}\\ 0_{13} & 0_{13} & e_{z}^{T} & 0_{13} & 0_{11} & -I_{1}\\ 2m_{\times } & 0_{33} & 0_{33} & 0_{33} & 0_{31} & 0_{31} \end{array}\right).$$

In A and C, the invariant quantities ${\hat{I}}_{\omega }$ and ${\hat{I}}_{a}$ are given in the following Equation (15):

(15) $$\begin{array}{c} {\hat{I}}_{\omega }=\hat{q}*\left(\omega -{\hat{b}}_{\omega }\right)*{\hat{q}}^{-1},\\ {\hat{I}}_{a}=\frac{1}{{\hat{s}}_{a}}\hat{q}*a*{\hat{q}}^{-1}. \end{array}$$

$I_{\times }$ is defined such that $I_{\times }u=I\times u$ for all $u\in R^{3}$.

It should be noticed that matrices A and C are constant if invariant quantities ${\hat{I}}_{\omega }$ and ${\hat{I}}_{a}$ are constant. ${\hat{I}}_{\omega }$ and ${\hat{I}}_{a}$ are constant if the UAV flies at a constant angular rate and there is constant acceleration in the NED coordinate frame. If these conditions are met, the IEKF becomes a linear Kalman filter, which guarantees convergence. This is one of the most distinctive features of the IEKF compared with the usual EKF. In addition, the differential equation for the quaternion estimation derived in Equation (6) intrinsically keeps the estimated quaternion normalized at all times. These advantageous features apply to all IEKFs, including the IEKF-av, which will be described in Section 3.2.

In case of usual EKF, the matrices A and C are derived from partial derivatives of process model and measurement model. Since the process model and measurement model are a nonlinear combination of attitude, angular rate, acceleration, bias in angular rate, and acceleration scale factor, the partial derivatives depend on those variables in a nonlinear manner, and vary with a change of those variables, which is not desirable for convergence. If all the variables are constant, the matrices A and C will be constant and the EKF also will work like a linear KF, assuring convergence. However, even though the UAV flies at a constant angular rate and constant acceleration in the NED coordinate frame, the matrices A and C are not constant, that is, nonlinear dynamics do not become linear, therefore the properties applicable to linear KF do not apply. On the contrary, in IEKF, the matrices A and C are constant in such a systematic way that A and C become constant if the UAV flies at a constant angular rate and constant acceleration in the NED coordinate frame as explained above. This advantage of IEKF over usual EKF is due to the use of invariance property in derivation of the IEKF [8,16].

Putting the above descriptions together, IEKF formulates the process model and measurement model to be invariant as Equations (4) and (5), thus making it possible to design the observer [estimator] to be invariant as Equation (6). Due to the invariance property of the observer (6), the sufficient condition for constant A and C becomes that invariant quantities ${\hat{I}}_{\omega }$ and ${\hat{I}}_{a}$ be constant. This sufficient condition for IEKF is relaxed compared with that for the usual EKF, for which attitude, angular rate, acceleration, bias in angular rate, and acceleration scale factor are required to be constant to keep A and C constant—thus formulating the process and measurement model in an invariant way and designing the observer to be invariant result in improved convergence property of the IEKF over the usual EKF [8].

In addition to the advantage of convergence property, the quaternion estimation $\hat{q}$ of the IEKF intrinsically keeps the unit norm constraint onto the quaternion. The magnitude $∥\hat{q}∥$ of the estimated quaternion is kept as a unit since the quaternion estimate obeys the differential equation presented at the first component of the observation Equation (6) [8], which is rephrased as Equation (16):

(16) $$\dot{\hat{q}}=\frac{1}{2}\hat{q}*\left(\omega -{\hat{b}}_{\omega }\right)+K_{q}E*\hat{q}.$$

The second term $K_{q}E*\hat{q}$ of Equation (16), which is multiplication of quaternion $\hat{q}$ with the vector $K_{q}E$, plays the role of preserving the magnitude of the quaternion constant [8]. In usual EKF, the corresponding term will be replaced by the multiplication of Kalman gain with measurement innovation. Therefore, the magnitude of the estimated quaternion deviates from the unit, thus invalid adjustment of the quaternion to rescale the magnitude to be a unit is inevitable. This deteriorates estimation performance of the usual EKF compared with the IEKF.

### 3.2. IEKF-av: Right IEKF for Estimation of Attitude and Velocity

This section describes the right IEKF used for estimating the attitude and velocity [8]. The state vector χ to be estimated consists of four variables: the attitude, velocity, bias in the angular rate measurement, and scale factor of the acceleration measurement (17):

(17) $$\chi ={\left(q,v,b_{\omega },s_{a}\right)}^{T}.$$

The process model for the state transition is the same as Equation (2), except that two equations for the location x and bias in the altitude measurement $b_{h}$ are deleted, resulting in Equation (18):

(18) $$\dot{\chi }=\left(\begin{array}{c} \dot{q}\\ \dot{v}\\ {\dot{b}}_{\omega }\\ {\dot{s}}_{a} \end{array}\right)=\left(\begin{array}{c} \frac{1}{2}q*\left(\omega -b_{\omega }\right)\\ g+\frac{1}{s_{a}}q*a*q^{-1}\\ 0\\ 0 \end{array}\right).$$

The measurements include the velocity from the GPS and the magnetic field from the magnetometer in the MEMS-AHRS as follows: $z={\left(z_{v},z_{m}\right)}^{T}$. Therefore, the measurement model is derived from that for the IEKF-lav (Equation (3)) by deleting the equations corresponding to the position and altitude measurements, resulting in Equation (19):

(19) $$z=\left(\begin{array}{c} z_{v}\\ z_{m} \end{array}\right)=\left(\begin{array}{c} v\\ q^{-1}*m*q \end{array}\right)$$

The inclusion of process noise is the same as that for the IEKF-lav, except that the equations for the states of the position and bias in the altitude measurement are deleted from Equation (2). Likewise, the noise incorporated measurement model is just Equation (5), except that the equations for $z_{x}$ and $z_{h}$ are deleted. The process model and measurement model for the IEKF-av are Equations (20) and (21):

(20) $$\dot{\chi }=\left(\begin{array}{c} \dot{q}\\ \dot{v}\\ {\dot{b}}_{\omega }\\ {\dot{s}}_{a} \end{array}\right)=\left(\begin{array}{c} \frac{1}{2}q*\left(\omega -b_{\omega }\right)\\ g+\frac{1}{s_{a}}q*a*q^{-1}\\ 0\\ 0 \end{array}\right)+\left(\begin{array}{c} M_{q}w_{q}*q\\ M_{v}w_{v}\\ q^{-1}*M_{b_{\omega }}w_{b_{\omega }}*q\\ s_{a}M_{s_{a}}w_{s_{a}} \end{array}\right),$$

(21) $$z=\left(\begin{array}{c} z_{v}\\ z_{m} \end{array}\right)=\left(\begin{array}{c} v+N_{v}\nu_{v}\\ q^{-1}*\left(m+N_{m}\nu_{m}\right)*q \end{array}\right).$$

The IEKF estimator for the attitude and velocity becomes the observer of Equation (22), and the corresponding error covariance P is determined by Equation (23):

(22) $$\dot{\hat{\chi }}=\left(\begin{array}{c} \dot{\hat{q}}\\ \dot{\hat{v}}\\ {\dot{\hat{b}}}_{\omega }\\ {\dot{\hat{s}}}_{a} \end{array}\right)=\left(\begin{array}{c} \frac{1}{2}\hat{q}*\left(\omega -{\hat{b}}_{\omega }\right)\\ g+\frac{1}{{\hat{s}}_{a}}\hat{q}*a*{\hat{q}}^{-1}\\ 0\\ 0 \end{array}\right)+\left(\begin{array}{c} K_{q}E*\hat{q}\\ K_{v}E\\ {\hat{q}}^{-1}*K_{b_{\omega }}E*\hat{q}\\ {\hat{s}}_{a}K_{s_{a}}E \end{array}\right),$$

(23) $$\dot{P}=AP+PA^{T}+MM^{T}-PC^{T}{\left(NN^{T}\right)}^{-1}CP,$$

where process error covariance M and measurement error covariance N are given in Equation (24):

(24) $$\begin{array}{c} M=Diag(M_{q},M_{v},M_{b_{\omega }},M_{s_{a}}),\\ N=Diag(N_{v},N_{m}). \end{array}$$

In Equations (22) and (23) for state χ and error covariance P, the Kalman gain K and output error E are given by Equations (25) and (26), respectively:

(25) $$K=-{\left(K_{q},K_{v},K_{b_{\omega }},K_{s_{a}}\right)}^{T}=PC^{T}{\left(NN^{T}\right)}^{-1},$$

(26) $$E=\left(\begin{array}{c} {\hat{z}}_{v}-z_{v}\\ m-\hat{q}*z_{m}*{\hat{q}}^{-1} \end{array}\right)=\left(\begin{array}{c} \hat{v}-v-N_{v}\nu_{v}\\ m-\hat{q}*q^{-1}*\left(m+N_{m}\nu_{m}\right)*q*{\hat{q}}^{-1} \end{array}\right).$$

To derive the linearized process model A and measurement model C, which are required for calculating the Kalman gain K and state error covariance P, the state error $\eta ={\left(\eta_{q},\eta_{v},\eta_{b_{\omega }},\eta_{s_{a}}\right)}^{T}$ is taken as Equation (27):

(27) $$\left(\begin{array}{c} \eta_{q}\\ \eta_{v}\\ \eta_{b_{\omega }}\\ \eta_{s_{a}} \end{array}\right)=\left(\begin{array}{c} \hat{q}*q^{-1}\\ \hat{v}-v\\ q*\left({\hat{b}}_{\omega }-b_{\omega }\right)*q^{-1}\\ \frac{{\hat{s}}_{a}}{s_{a}} \end{array}\right).$$

Linearizing the state error differential system and fitting the linearized model to Equation (12) yields matrices A and C, as shown in Equations (28) and (29) [8], respectively:

(28) $$A=\left(\begin{array}{cccc} 0_{33} & 0_{33} & -\frac{1}{2}I_{3} & 0_{31}\\ -2{\hat{I}}_{a\times } & 0_{33} & 0_{33} & -{\hat{I}}_{a}\\ 0_{33} & 0_{33} & {\hat{I}}_{\omega \times } & 0_{31}\\ 0_{13} & 0_{13} & 0_{13} & 0_{11} \end{array}\right),$$

(29) $$C=\left(\begin{array}{cccc} 0_{33} & I_{3} & 0_{33} & 0_{31}\\ 2m_{\times } & 0_{33} & 0_{33} & 0_{31} \end{array}\right).$$

### 3.3. ecl-EKF for Estimation of Attitude, Velocity, and Location

An EKF for estimating the attitude, velocity, and location, called the ecl-EKF, is used for comparison purposes [23]. The ecl-EKF is an EKF-based navigation algorithm that was developed by the PX4 project [22]. The ecl-EKF sets a state vector encompassing the following 24 state variables: the attitude by quaternion, velocity, position, IMU delta angle bias, IMU delta velocity bias, earth magnetic field, magnetometer bias, and horizontal wind velocity. It uses measurements of the angular rate and acceleration by the IMU, along with the GPS position, GPS velocity, pressure altitude, and geomagnetic field.

The ecl-EKF uses an output predictor and data buffer to fuse data from sensors with different time delays and data rates. The EKF interacts with the strap down inertial navigation unit, data buffer, and output predictor. The ecl-EKF is capable of fusing a large range of different sensor types. It also detects outliers in sensor measurements. The ecl-EKF code can be found at GitHub [24]. For the implementation and details of the ecl-EKF and PX4, please refer to the documents on PX4 at the site [25].

## 4. Implementation for Navigation of UAVs

**Figure 1:**
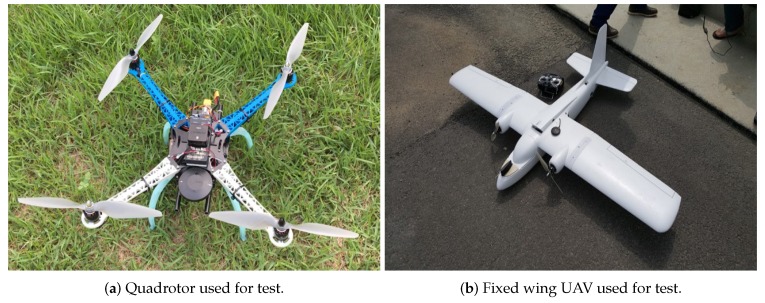
Unmanned aerial vehicles (UAVs) used to test methods.

The IEKF methods and ecl-EKF were implemented for the navigation of a four-rotary wing UAV, otherwise called a quadrotor, and a fixed wing UAV. The fixed wing UAV was used to test the IEKF-lav and ecl-EKF, and the quadrotor was used to test the IEKF-av and ecl-EKF. Figure 1 shows the quadrotor and fixed wing UAV used for the flight test. Each UAV had a global navigation satellite system (GNSS), an AHRS, and a barometric altimeter. Table 1 and Table 2 explain the UAVs used for the tests, and Table 3 lists the performance of the GNSS used in the experiments.

The two UAVs use the same flight control unit of Pixhawk v2 (3DR, Berkeley, CA, US) [26]. Pixhawk v2 has an internal accelerometer, gyroscope, and barometer, in addition to a CPU and external magnetometer. The components are shown in Table 4. The gyroscope can measure up to 2000 deg/s of angular rate in three axes with maximum output data rate 8000 Hz, root mean square (RMS) noise 0.1 ${}^{\circ }/s$, nonlinearity ±0.1%, and noise spectral density 0.01 ${}^{\circ }/s/\sqrt{\mathrm{Hz}}$. The accelerometer measures three axes’ acceleration at the maximum output data rate of 4000 Hz, with full scale range of ±16 g , nonlinearity ±0.5%, RMS noise 8 mg , and noise power spectral density $300\;\mu g/\sqrt{\mathrm{Hz}}$. The barometer measures atmospheric pressure, which is transformed to height. Its measurement range is 10 to 1200 mbar, with accuracy of ±1.5 mbar at 25 ${}^{\circ }C$ and 750 mbar atmosphere. Its error band is ±2.5 mbar at −20 ${}^{\circ }C$ to +85 ${}^{\circ }C$ temperature and a 450 to 1100 mbar environment. The magnetometer measures magnetic field strength in three axes, with the measurement range of ±8 Gauss, linearity ±0.1% of full scale at ±2.0 Gauss input range, hysteresis of ±25 ppm at ±2.0 Gauss input range, at the maximum output rate of 220 Hz.

**Figure 2:**
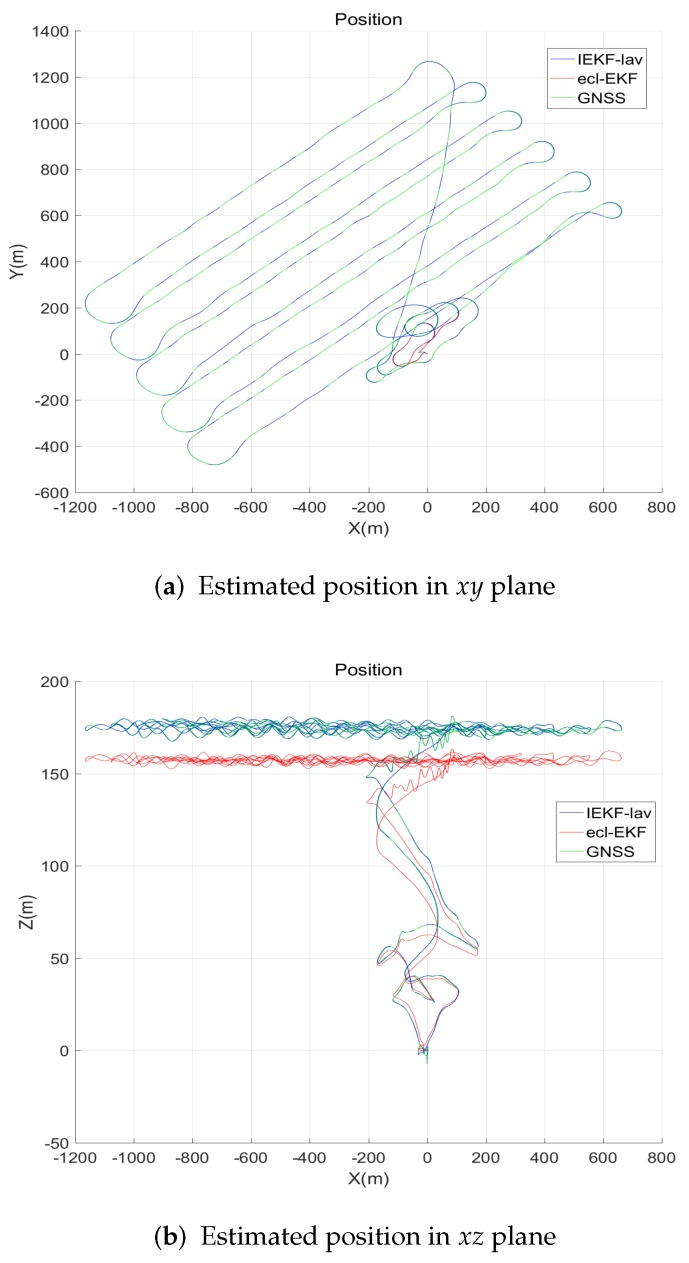
Robot positions estimated by invariant extended Kalman filter (IEKF)-lav and ecl-EKF.

The flight times were 700 s and 1800 s for the fixed wing UAV and quadrotor, respectively. The trajectories of the flights will be shown in the next sections, along with the estimation results (Figure 2).

The UAVs flew outdoors in open air, so that there was no interference to GNSS reception. The UAVs were remotely controlled using a dedicated joystick. The estimated states are not used for control of the UAVs. All the flight data including the measured sensor data are stored on board the flight control unit. Using the stored measurement data, the methods are applied offline for comparison. The methods do not run in real-time and are implemented using Matlab version R2018a (MathWorks, Natick, MA, US) on Windows 10 Pro desktop computer with CPU Intel(R) Core(TM) (Intel, Santa Clara, CA, US) i7-7700k CPU @4.20 GHz 4.20 GHz. For the IEKF-lav, each iteration takes a computation time of 0.084 ms. The computation time is less than and negligible compared to the IMU measurement update rate of 0.02 s within which the IEKF should iterate for possible real-time application.

In the experiments, measurement update rate of each sensor is as follows: data from AHRS-acceleration, angular rate, and magnetic field are measured at every 0.02 s, while GNSS velocity and position are sampled at every 0.2 s, and barometric altitude at every 0.1 s. IEKF is implemented to run at the rate of 0.02 s in synchronization with the time of AHRS measurement. For the other measurements, such as the position and velocity from GNSS and barometric altitude which are sensed at different rates from the AHRS measurement rate, the previous measurement data are used when there were no available measurement data at every iteration of the IEKF. One of the other possible methods is that whenever each measurement is available, a correction step per the available measurement is executed, which is the method used for ecl-EKF.

Application of IEKF and ecl-EKF requires tuning of parameter values. The process error covariance M and measurement error covariance N in Equations (8) and (24) affect the performance of the IEKF. They should be adjusted for each application depending on the sensor performance and system characteristics determining the process model Equations (4) and (18) and measurement model Equations (5) and (19). In our formulation, since the process model and measurement model are involved with the sensor measurements, they are mostly dependent on the sensor measurement performance. Since the IEKF-lav and IEKF-av use the same sensors and control unit, the parameter values for M and N are set to be the same. Considering the degree of uncertainty of each sensor measurement, and to result in the best performance in the sense that there is lower measurement innovation and faster convergence of the internal variables, the error covariance are set to Equation (30), through trial and error, to the best of the authors’ effort:

(30) $$\begin{array}{c} M_{q}=M_{v}=M_{x}=M_{b_{\omega }}=Diag\left(0.1,0.1,0.1\right),\;M_{s_{a}}=M_{b_{h}}=0.1,\\ N_{v}=Diag\left(0.5,0.5,0.5\right),\;N_{x}=Diag\left(0.1,0.1,0.1\right),\;N_{h}=0.1,\;N_{m}=Diag\left(0.1,0.1,0.1\right). \end{array}$$

## 5. Discussion of Implementation Results

Two major comparisons of the results were made: (1) between the IEKF-lav and ecl-EKF for estimates of the location, attitude, and velocity, and (2) between the IEKF-av and ecl-EKF for the estimations of the attitude and velocity. The estimated state variables, Kalman gain, state error covariance, and measurement innovation were compared where appropriate.

The first comparison highlighted the distinctive properties of the IEKF relative to the usual EKF. The second comparison underscored the performance of the IEKF without location and altitude measurements compared with the EKF, for which location and altitude measurements were used. It showed that, even though there were no location and altitude measurements, the IEKF-av produced estimates of the velocity and attitude that were comparable to those of the ecl-EKF.

### 5.1. Comparison between IEKF-lav and ecl-EKF

**Figure 3:**
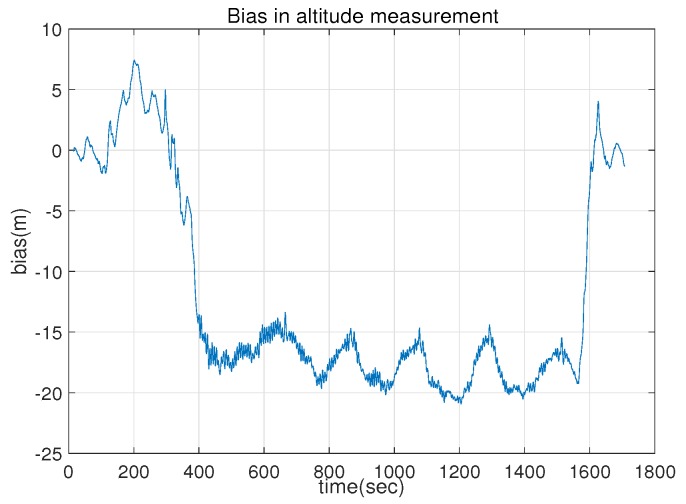
Estimated bias in barometric altitude measurement by IEKF-lav.

Figure 2 compares the position estimates of the IEKF-lav and ecl-EKF for the flight of the fixed wing UAV. The IEKF-lav and ecl-EKF yielded similar position estimations on the xy plane, while the xz plane position estimates showed a difference. Although there was no definite evidence for which estimate was more accurate between the *z* directional location estimations of the IEKF-lav and ecl-EKF, the difference between the elevation estimates could be explained by the estimation of bias in the barometric altitude measurement. The IEKF-lav estimated the bias in the altitude measurement by the barometric altimeter, while the ecl-EKF does not. Figure 3 shows that the IEKF-lav estimated the bias in the altitude measurement to be approximately 15–20 m at 400–1600 s after the flight began. It should be noted that the difference between the altitude estimates of the IEKF-lav and ecl-EKF, as shown in Figure 2, corresponds approximately to the estimated bias in the barometric measurement of the IEKF-lav.

The difference between the altitudes estimated by IEKF-lav and the ecl-EKF corresponds to the bias estimated by the IEKF-lav, since the ecl-EKF neither estimates nor calibrates the bias while IEKF-lav does. Barometric pressure is transformed to the altitude. To get a more accurate altitude from the measured barometric pressure, it is required to calibrate the transformed altitude taking the local atmospheric pressure into account. However, the experiment uses the transformed altitude without the calibration. Therefore, estimating the error and correcting the altitude is desirable since the transformed altitude differs from the true altitude. The IEKF regards the error as the bias in altitude measurement. The bias is estimated and corrected using the altitude which is represented in state, as indicated by the third row of the measurement model Equation (3). It is shown as Equation (31):

(31) $$z_{h}=x\cdot e_{z}+b_{h}.$$

In Equation (31), the term $x\cdot e_{z}$ represents the altitude element of the location state x. The bias $b_{h}$ is the difference between the altitude $z_{h}$ transformed from barometric pressure measurement and the altitude $x\cdot e_{z}$. If x is estimated as $\hat{x}$, the bias is the difference between the estimated altitude and the measured altitude. Since ecl-EKF does not take the bias into account, the altitude estimated by ecl-EKF differs from the altitude estimated by IEKF-lav, by the amount corresponding to the estimated bias.

**Figure 4:**
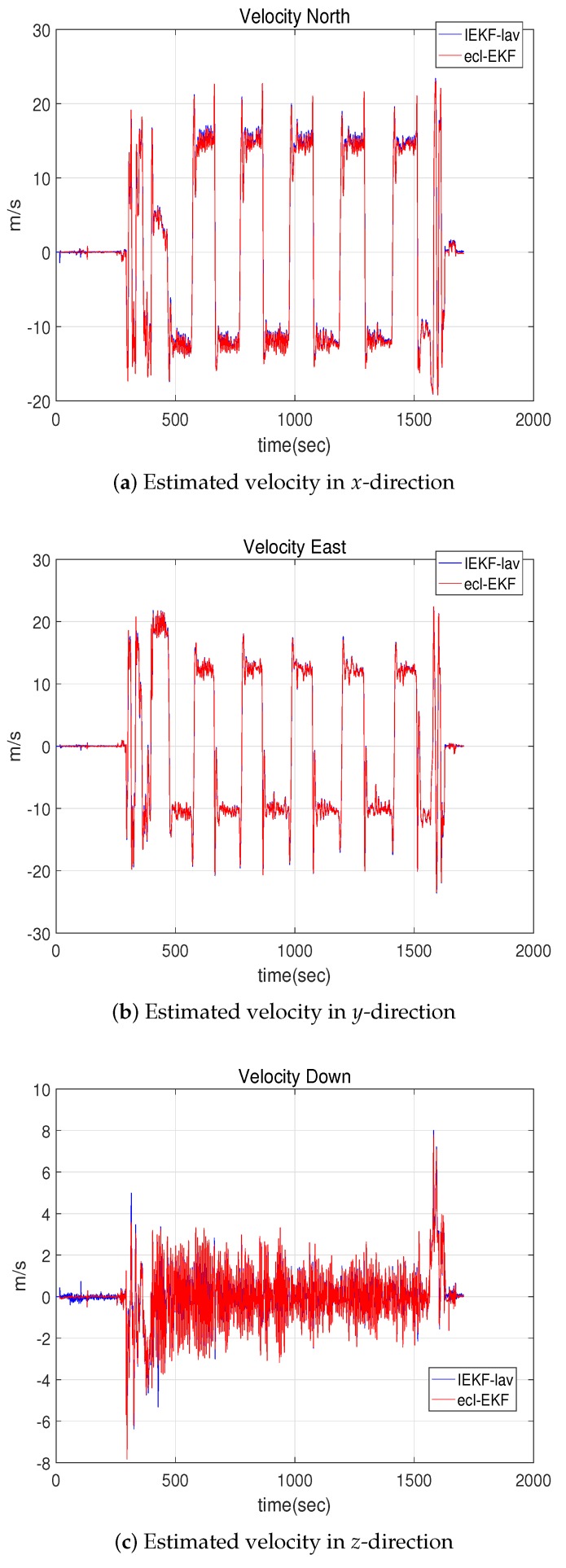
Velocities estimated by IEKF-lav and ecl-EKF.

**Figure 5:**
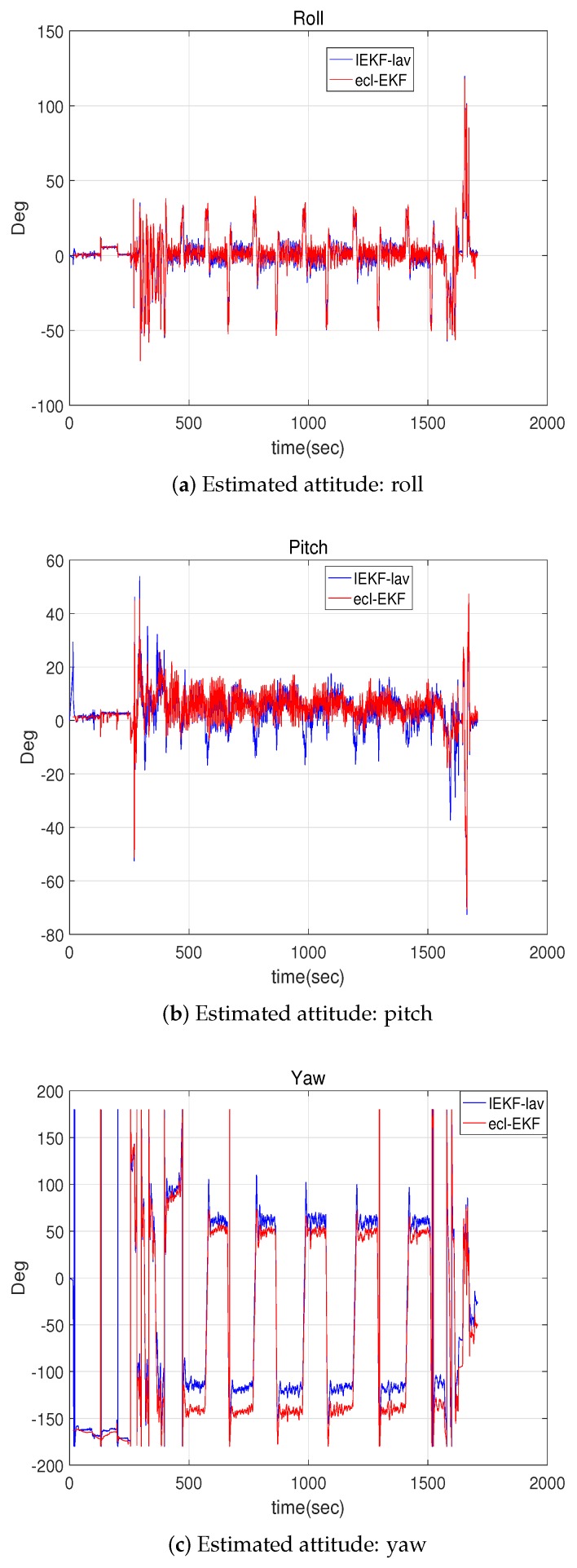
Attitude represented by Euler angles estimated by IEKF-lav and ecl-EKF.

Figure 4 and Figure 5 show the estimates of the velocity and attitude. The estimates by the IEKF-lav and ecl-EKF are similar, and there is no clear indication of which estimate is better than the other, as is the case for the comparison of the position estimations shown in Figure 2.

**Figure 6:**
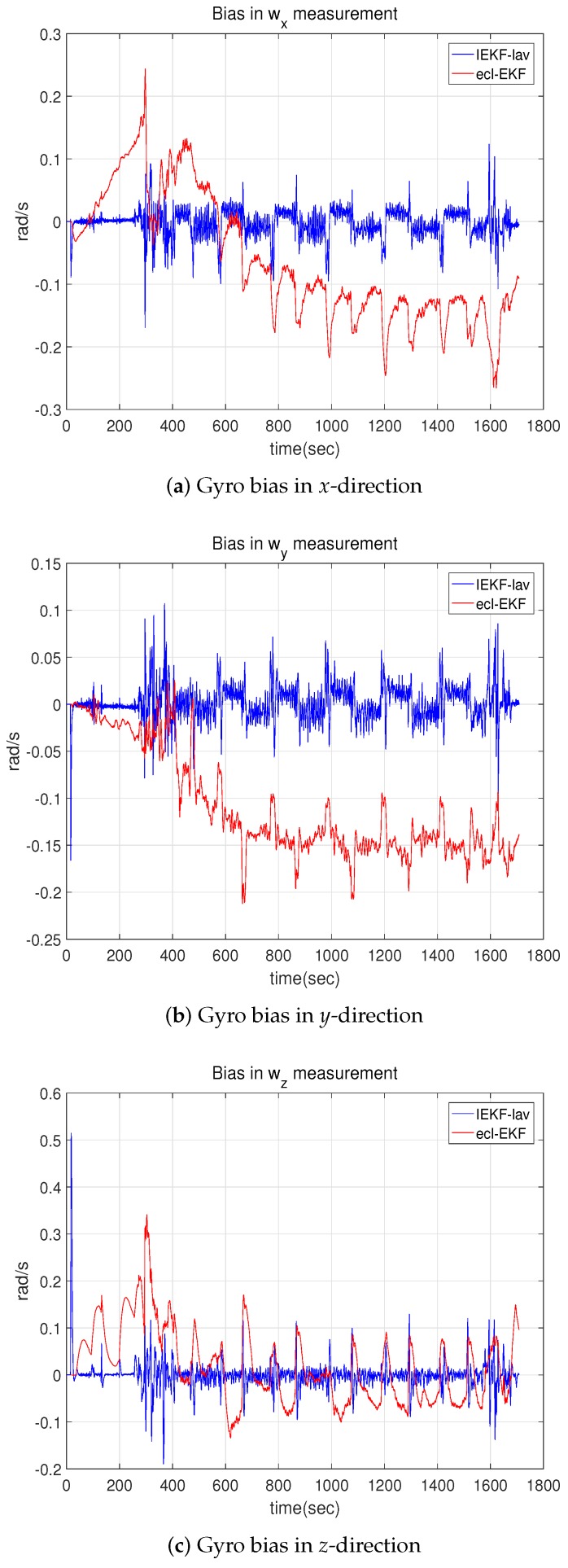
Bias in angular rate measurement by gyro estimated by IEKF-lav and ecl-EKF.

Figure 6 depicts the estimated bias in the angular rate measurement by the gyroscope. Unlike the estimation results for the velocity, attitude, and position in the xy plane, the estimations of the bias in angular rate show a difference. The bias estimated by the IEKF-lav varies less over time than does the bias by the ecl-EKF. This is one of the distinctive features of the IEKF compared with the EKF. The estimation results and internal parameters of the IEKF are more convergent than those of the EKF.

**Figure 7:**
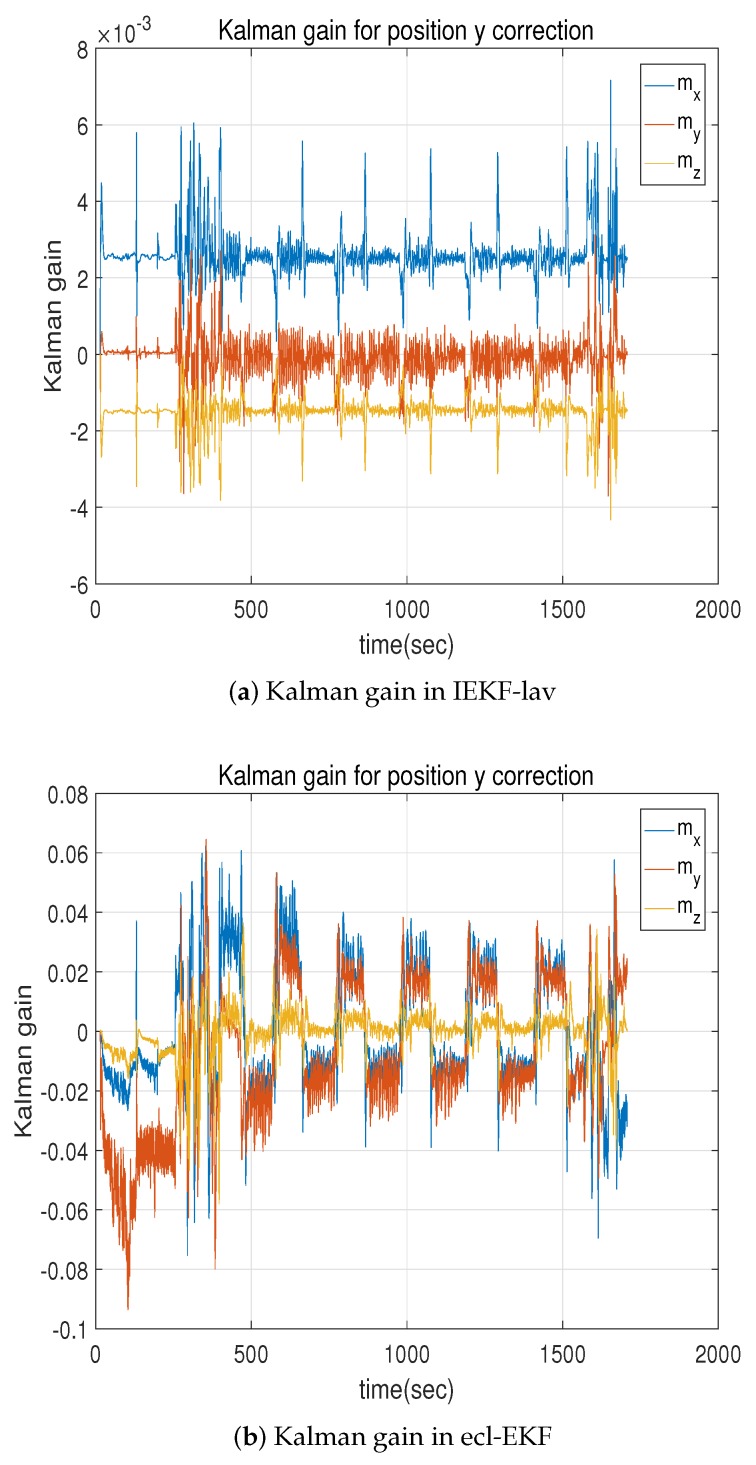
Comparison of Kalman gains for correction of *y*-coordinate position relative to magnetic field measurement innovation.

Figure 7 compares the change in the Kalman gain by the IEKF-lav and ecl-EKF. The Kalman gain by the ecl-EKF changes periodically, as shown in Figure 7b. A comparison of Figure 5 and Figure 7b shows that the change in the Kalman gain occurs in the ecl-EKF case when the attitude changes during the flight. In contrast, the Kalman gain of the IEKF-lav does not change with a change in the heading, even though it also suffers from high-frequency jitter just like the ecl-EKF. This is the most salient feature of the IEKF compared with the EKF. Matrices A and C expressed by Equations (13), (14), (28), and (29) depend on invariant quantities ${\hat{I}}_{\omega }$, ${\hat{I}}_{a}$, and geomagnetic field m appropriate for a given local space. If the invariant quantities ${\hat{I}}_{\omega }$, ${\hat{I}}_{a}$, and geomagnetic field m remain constant, matrices A and C are also constant. Thus, the resulting Kalman gain K and state error covariance P converge to constants. As a whole, although it is not clearly verifiable that the estimated state variables for the IEKF-lav are better than those for the ecl-EKF, the analysis of the internal parameters such as the Kalman gain shows that the convergence property of the IEKF-lav outperforms that of the ecl-EKF.

Table 5 compares the convergence property of the Kalman gain numerically. The table lists the values for the mean, standard deviation, and ratio between the standard deviation and mean of the Kalman gain for the IEKF-lav and ecl-EKF.

To clarify the elements of Kalman gain matrix to be compared, each element of the Kalman gain (9) is represented as $K_{x/z}$, as shown in Equation (32):

(32) K=Kq0/zvxKq0/zvyKq0/zvzKq0/zx⋯Kq0/zmzKqx/zvxKqx/zvyKqx/zvzKqx/zx⋯Kqx/zmzKqy/zvx···⋯Kqy/zmz⋮⋮Kbh/zvxKbh/zvyKbh/zvzKbh/zx⋯Kbh/zmz=Kx/zx∈X,z∈Z,whereX={q0,qx,qy,qz,vx,vy,vz,x,y,z,bwx,bwy,bwz,sa,bh},Z={zvx,zvy,zvz,zx,zy,zz,zh,zmx,zmy,zmz}.

Here, the Kalman gain $K_{x/z}$ refers to the gain for the correction of the state x with respect to the innovation in the measurement z. Table 5 lists the statistics for nine elements of the Kalman gain matrix. It shows that, out of the nine standard deviation to mean ratio (SM ratio) values, seven of them for the IEKF-lav are less than those for the ecl-EKF. Only for the two Kalman gains $K_{q_{y}/z_{m_{y}}}$ and $K_{y/z_{m_{y}}}$, the SM ratios for the ecl-EKF are less than those for the IEKF-lav. Thus, it can be concluded that the Kalman gains for the IEKF-lav are more convergent than those for the ecl-EKF.

**Figure 8:**
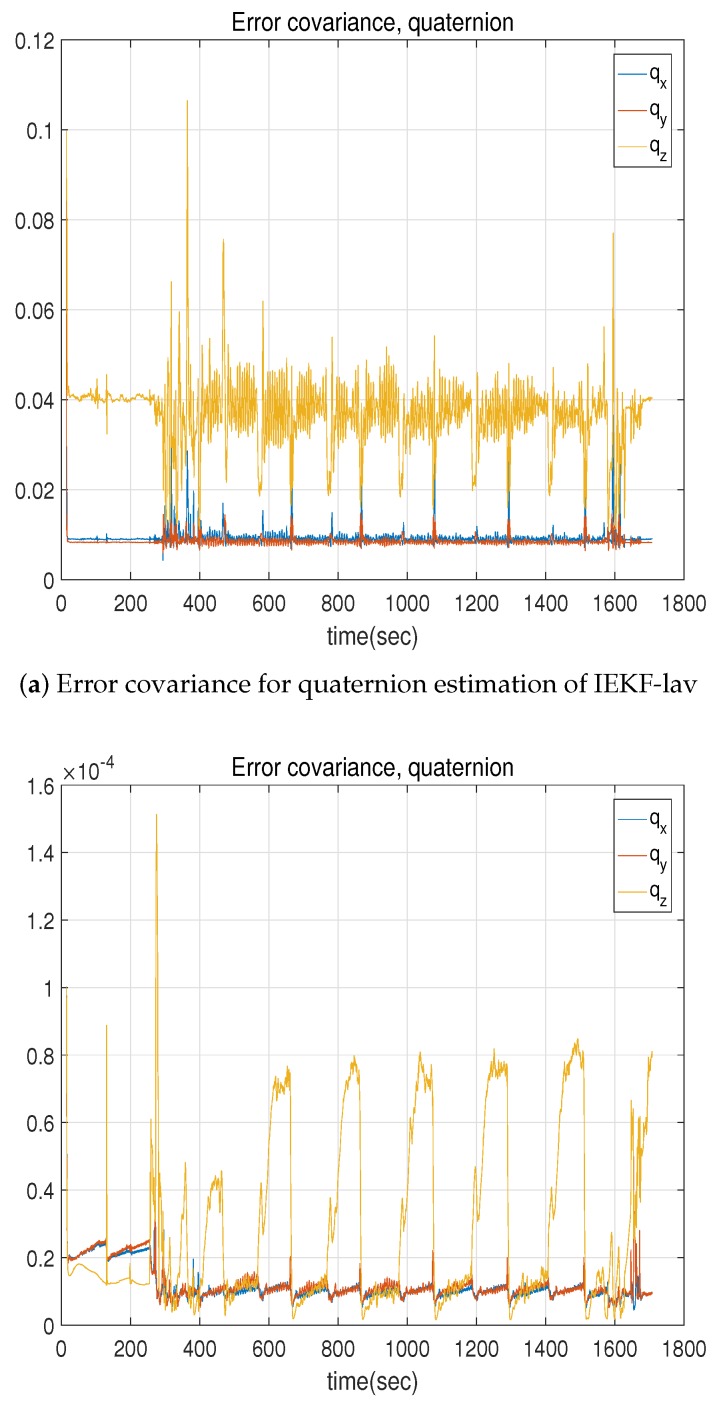
Comparison of error covariance for quaternion estimation between IEKF-lav and ecl-EKF.

There was also a crucial covariance difference between the IEKF-lav and ecl-EKF. Figure 8 compares the covariance for the state of the quaternion variables ${\left(q_{x}\left(t\right),q_{y}\left(t\right),q_{z}\left(t\right)\right)}^{T}$. The variation of the covariance with time for the IEKF-lav is less than that for the ecl-EKF. Table 6 lists the results of a statistical analysis of the variation with time based on the index of the SM ratio, as was used for the Kalman gain comparison. Both Figure 8 and Table 6 indicate that the convergence of the covariance for the IEKF-lav outperforms that for the ecl-EKF. This was due to the same justification as in the case for the convergence of the Kalman gain.

**Figure 9:**
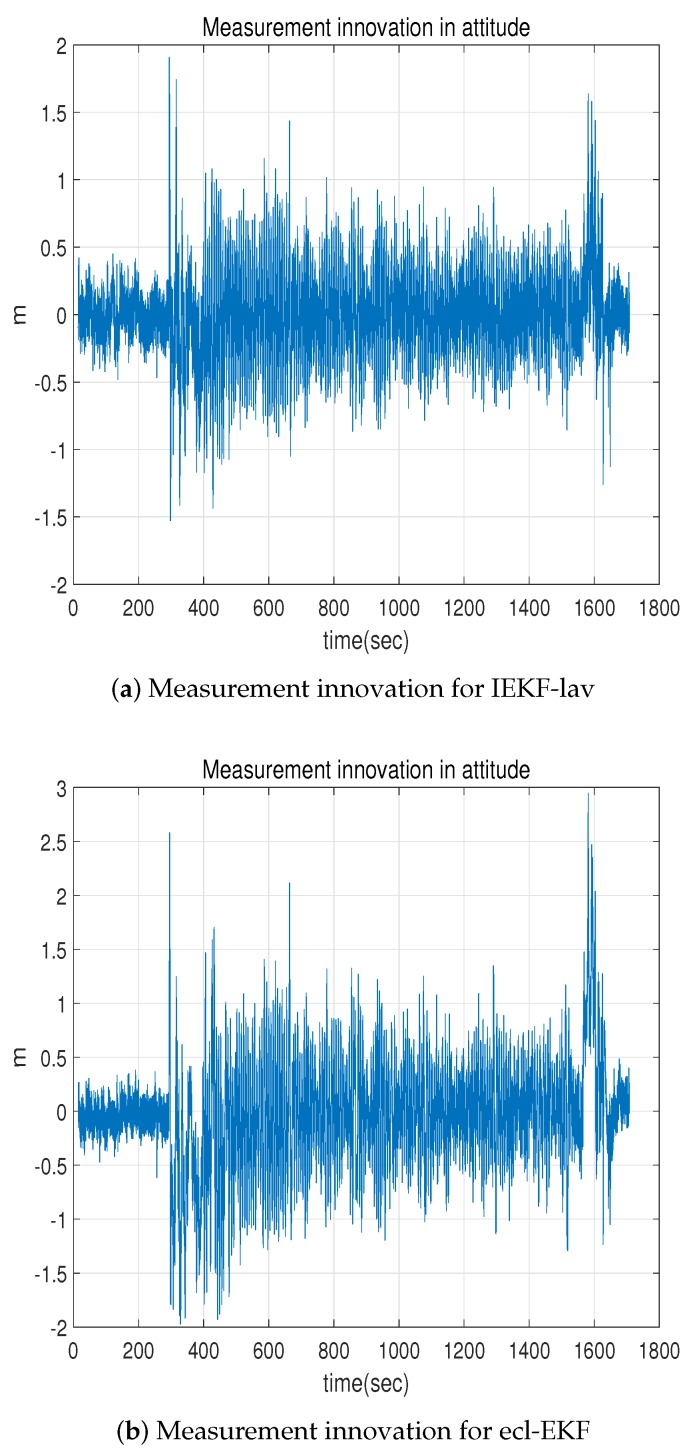
Comparison of measurement innovation for altitude (*z*-coordinate position).

Figure 9 shows the measurement innovation for the altitude. The mean and standard deviation of the measurement innovation for the IEKF-lav are −0.0012 m and 0.3147 m, while those for the ecl-EKF are −0.0333 m and 0.5147 m in Figure 9, respectively. As can be expected from the position estimation result in the *z*- direction (Figure 2b), the measurement innovation for the ecl-EKF is a little larger than that for the IEKF-lav.

For the IEKF-lav, each iteration takes the computation time of 0.084 ms. The computation time is less than and negligible compared to the IMU measurement update rate of 0.02 s within which the IEKF should iterate for possible real-time application. One iteration of ecl-EKF takes 4.3 ms which is also short enough for real-time application compared to the IMU measurement rate. The computation time for ecl-EKF is 51 times longer than that for IEKF-lav. However, for the sake of fair comparison, it should be noticed that the ecl-EKF method includes calibration of IMU data before application of EKF and it estimates state variables such as bias of delta angle, bias of delta velocity, earth magnetic field, and horizontal wind velocity which are not estimated in IEKF-lav. Therefore, it can be concluded that the computation time given above suggests that IEKF-lav is computationally better than or at least comparable with the usual EKF, though the figures given above are not absolute ones applicable to general IEKF and usual EKF.

### 5.2. Comparison between IEKF-av and ecl-EKF

**Figure 10:**
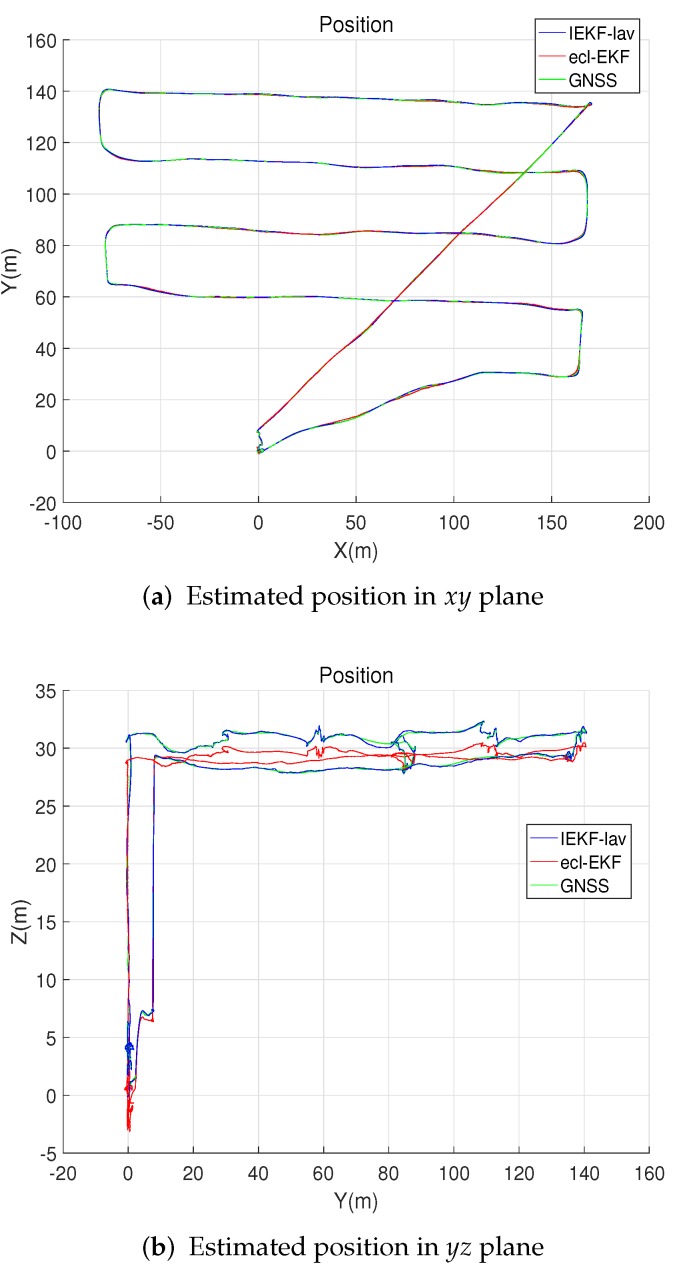
Robot positions estimated by IEKF-lav and ecl-EKF for quadrotor flight.

This section compares the IEKF-av and ecl-EKF based on the estimation results for the attitude and velocity during the quadrotor flight test. The IEKF-av does not use position and altitude measurements, while the ecl-EKF does use them. Although an analysis of the IEKF-lav is not intended in this section, the flight trajectory estimated by the IEKF-lav is shown in Figure 10 for the purpose of easily understanding the test conditions. The change in attitude can be determined from Figure 10.

**Figure 11:**
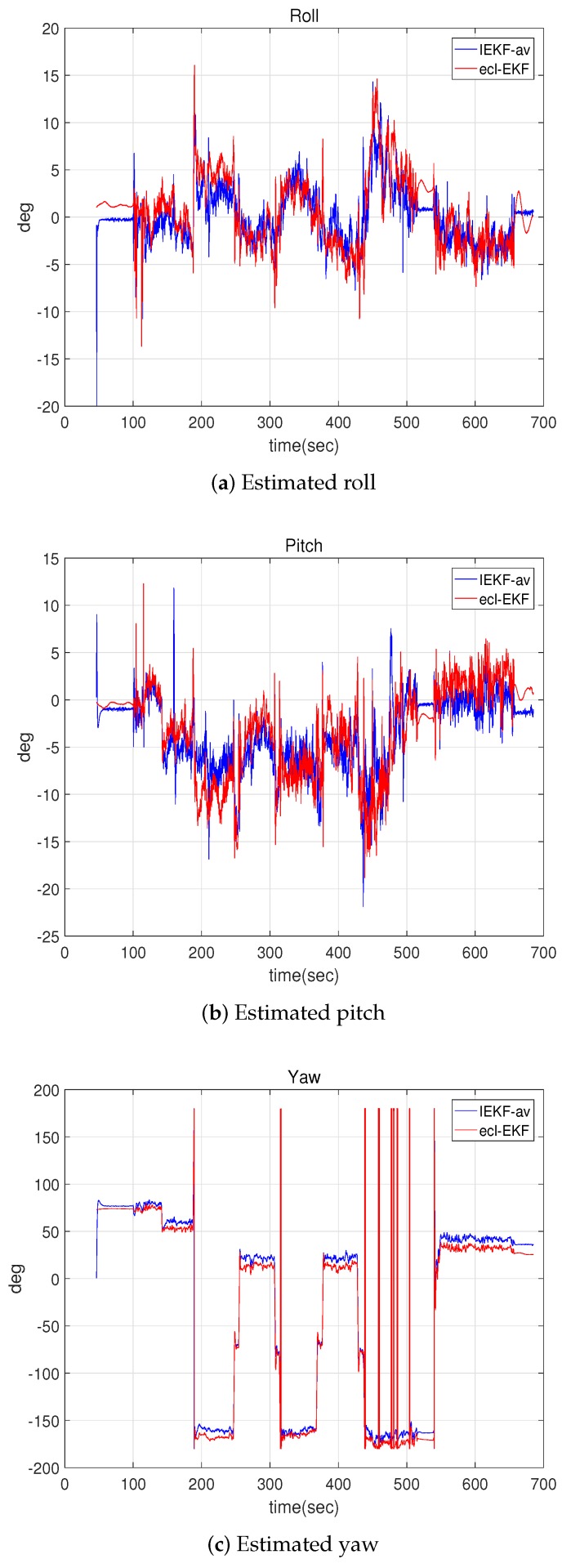
Attitudes estimated by IEKF-av and ecl-EKF for quadrotor flight test.

**Figure 12:**
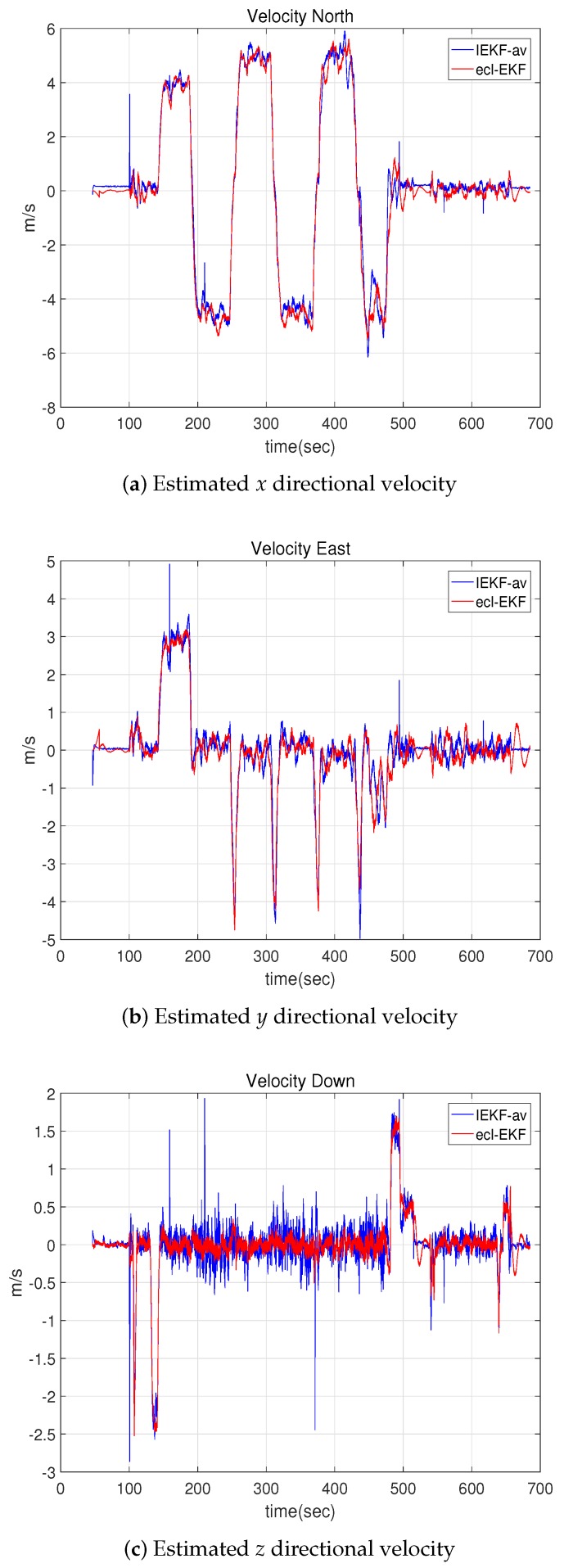
Velocities estimated by IEKF-av and ecl-EKF for quadrotor flight test.

Figure 11 shows the attitude estimations for the IEKF-av and ecl-EKF, and Figure 12 shows the velocity estimations. As suggested by the location, attitude, and velocity estimation results for the IEKF-lav and ecl-EKF, the IEKF-av and ecl-EKF have comparable attitude and velocity estimation results. Although it is not possible to say which is better, this verifies that the IEKF-av works well without location and altitude measurements.

**Figure 13:**
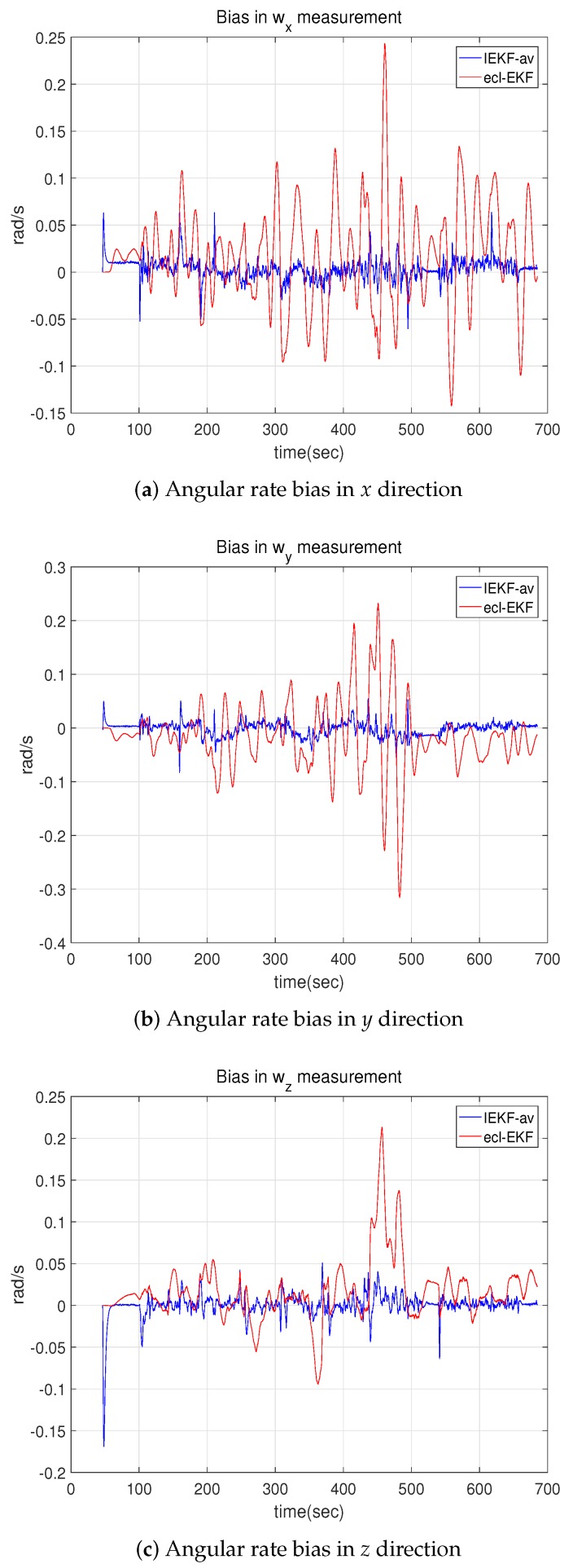
Bias values estimated for angular rate measurements by IEKF-av and ecl-EKF.

The bias estimations for the angular rate measurements showed a distinctive difference, and Figure 13 compares the estimated bias values. While the bias in the angular rate measurement for the fixed wing UAV flight shows a difference in the average magnitude, as shown in Figure 6 of Section 5.1, the bias in the quadrotor flight test shows a difference in the degree of fluctuation. The bias for the ecl-EKF shows a larger fluctuation than that for the IEKF-av, as shown in Figure 13.

**Figure 14:**
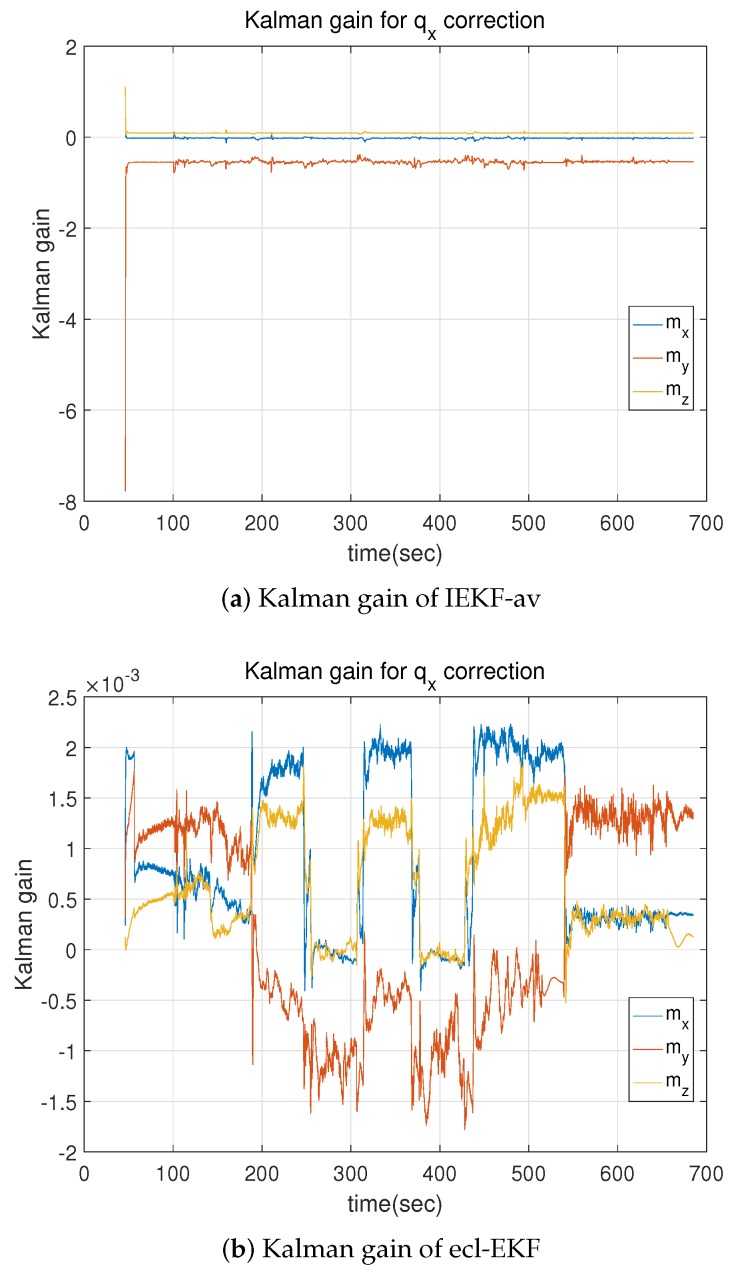
Comparison of Kalman gain for correction of *x*-component of quaternion relative to innovation of magnetic field measurements of IEKF-av and ecl-EKF.

The Kalman gain and state error covariance most critically discriminate the IEKF-av from the ecl-EKF. Only the Kalman gain is analyzed to save space. As shown in Figure 14, the IEKF-av has a more convergent Kalman gain, while that of the ecl-EKF exhibits periodic fluctuation, which depends on the attitude change in the quadrotor, like the results shown in Section 5.1.

Table 7 lists the results of a numerical analysis of the Kalman gain fluctuation. For all the Kalman gains except the Kalman gain $K_{b_{\omega_{y}}}$ with respect to $z_{m_{y}}$, the SM ratios for the IEKF-av are less than those for the ecl-EKF. Most of the Kalman gains for the IEKF-av are more convergent than those for the ecl-EKF. This is the same feature as already revealed by Table 5 in Section 5.1.

## 6. Conclusions

This paper evaluated the estimation performance and revealed the properties of the IEKF-based navigation method through flight experiments with UAVs, and particularly through a comparison with the ecl-EKF, which is one of the prevalent navigation filters for small UAVs with limited sensor measurements and computational capacity.

One of the distinctive features of the IEKF is its convergent Kalman gain, state error covariance, and estimation parameters such as measurement innovation and bias estimation. This is because the linearized process model and measurement model of the IEKF depend only on invariant quantities, and the invariant quantities depend on the angular rate and acceleration. If the angular rate and acceleration are constant, the invariant quantities are constant, and thus the linearized model is constant and the filter converges to a linear Kalman filter. Even if the angular rate or acceleration changes instantaneously, the Kalman gain and state error covariance converge again soon after the change. This property was verified in the Section 5.1 and Section 5.2. Section 5.2 also demonstrated that, without location and altitude measurements, the IEKF was able to yield attitude and velocity estimations that were comparable to the estimations by the ecl-EKF for which location and altitude measurements were utilized.

The IEKF can be derived using either the abstract Lie group methodology or matrix Lie group methodology [1,7]. This paper used the abstract Lie group-based derivation. For further research, it is recommended to derive the matrix Lie group-based formulation of the IEKF for the same problem considered in this paper. In addition, it is expected that the problems addressed through the matrix Lie group approach [7] can be solved by the abstract Lie group-based approach in the subsequent research.

## Figures and Tables

**Table 1:** Quadrotor used for navigation test.

Parameter	Specification
Model	S500 Glass Fiber Quadrotor Frame 480 mm
Distance between motor center	480 mm
Weight, frame only	405 g
Height, frame only	170 mm
Flight controller	Pixhawk v2

**Table 2:** Fixed wing unmanned aerial vehicles (UAV) used for navigation test.

Parameter	Specification
Model	ALBATROSS *
Wingspan	1800 mm
Weight (camera and battery included)	4.0 kg
Flight controller	Pixhawk V2

* This is different from the Albatross produced by the company Applied Aeronautics.

**Table 3:** Global navigation satellite system (GNSS) used for the navigation test.

Parameter	Specification
Manufacturer	u-blox
Model	NEO-M8P
GNSS	GPS, GLONASS, BeiDou
Grade	Professional
Accuracy of time pulse signal	RMS 30 ns; 99% 60 ns
Velocity accuracy	0.05 m/s
Max navigation update rate	Raw 10 Hz
Horizontal position accuracy *	Standalone 2.5 m CEP; RTK 0.025 m + 1 ppm CEP

* Standalone mode is used in the test.

**Table 4:** Composition of flight control unit, Pixhawk v2.

Component	Model	Maker
CPU	STM32F427; flash 2MiB, RAM 256KiB	PX4 Team
Accelerometer, gyroscope	MPU9250	InvenSense Inc.
Barometer	MS5611	TE Connectivity company
External Magnetometer	HMC5983	Honeywell

**Table 5:** Comparison of Kalman gain for estimation of location, attitude, and velocity in fixed wing flight test.

Kalman Gain Element	IEKF-lav			ecl-EKF		
	$\mu_{K}$	$\sigma_{K}$	$\frac{\sigma_{K}}{|\mu_{K}|}$	$\mu_{K}$	$\sigma_{K}$	$\frac{\sigma_{K}}{|\mu_{K}|}$
$K_{q_{y}/z_{m_{x}}}$	0.6665	0.0970	0.1455	$-5.4978\times 10^{-4}$	0.0010	1.8199
$K_{q_{y}/z_{m_{y}}}$	0.0100	0.0974	9.7085	$-3.4738\times 10^{-4}$	0.0011	3.2052
$K_{q_{y}/z_{m_{z}}}$	−0.3907	0.0562	0.1438	$7.2672\times 10^{-4}$	0.0010	1.4262
$K_{v_{x}/z_{m_{x}}}$	−3.8793	0.4596	0.1185	−0.0019	0.0311	16.5717
$K_{v_{x}/z_{m_{y}}}$	−0.3273	0.6017	1.8383	−0.0092	0.0331	3.6085
$K_{v_{x}/z_{m_{z}}}$	2.3121	0.2638	0.1141	$-8.3181\times 10^{-4}$	0.0117	14.0127
$K_{y/z_{m_{x}}}$	0.0026	$6.1468\times 10^{-4}$	0.2370	$-8.0811\times 10^{-4}$	0.0204	25.2135
$K_{y/z_{m_{y}}}$	$-1.0957\times 10^{-4}$	$5.6941\times 10^{-5}$	5.1970	−0.0079	0.0243	3.0778
$K_{y/z_{m_{z}}}$	−0.0015	$3.919\times 10^{-5}$	0.2614	$-3.4259\times 10^{-4}$	0.0075	22.0134

**MI**: Measurement innovation; $\mu_{K}$: mean of Kalman gain; $\sigma_{K}$: standard deviation of Kalman gain; $\frac{\sigma_{K}}{|\mu_{K}|}$: standard deviation to mean ratio (SM ratio).

**Table 6:** Comparison of error covariance for estimation of location, attitude, and velocity in fixed wing flight test.

State	Variable	IEKF-lav			ecl-EKF		
		$\mu_{C}$	$\sigma_{C}$	$\frac{\sigma_{C}}{\mu_{C}}$	$\mu_{C}$	$\sigma_{C}$	$\frac{\sigma_{C}}{\mu_{C}}$
Quaternion	$q_{x}$	0.0094	0.0024	0.2523	$1.1568\times 10^{-5}$	$4.9706\times 10^{-6}$	0.4297
	$q_{y}$	0.0086	0.0012	0.1349	$1.2188\times 10^{-5}$	$5.3028\times 10^{-6}$	0.4351
	$q_{z}$	0.0369	0.0076	0.2073	$2.9485\times 10^{-5}$	$2.5857\times 10^{-5}$	0.8769
Velocity	$v_{x}$	0.6990	0.0534	0.0764	0.0161	0.0047	0.2893
	$v_{y}$	0.7653	0.0490	0.0640	0.0162	0.0044	0.2750
	$v_{z}$	0.5161	0.0568	0.1100	0.0093	0.0024	0.2611
Position	*x*	0.0100	$6.5039\times 10^{-4}$	0.0649	0.0563	0.0211	0.3752
	*y*	0.0100	$6.5039\times 10^{-4}$	0.0649	0.0564	0.0212	0.3757
	*z*	0.0090	$5.6567\times 10^{-4}$	0.0629	0.0673	0.0098	0.1457
Bias in	$\omega_{x}$	0.0271	0.0023	0.0841	$1.7199\times 10^{-10}$	$2.8369\times 10^{-11}$	0.1649
angular	$\omega_{y}$	0.0268	0.0021	0.0798	$1.7036\times 10^{-10}$	$2.4400\times 10^{-11}$	0.1432
rate	$\omega_{z}$	0.0357	0.0031	0.0859	$2.6572\times 10^{-10}$	$3.4483\times 10^{-11}$	0.1298

$\mu_{C}$: mean of covariance; $\sigma_{C}$: standard deviation of covariance; $\frac{\sigma_{C}}{\mu_{C}}$: standard deviation to mean ratio (SM ratio).

**Table 7:** Comparison of Kalman gain for estimation of attitude and velocity in quadrotor flight test.

Kalman Gain Element	IEKF-av			ecl-EKF		
	μ	σ	$\frac{\sigma }{\left|\mu \right|}$	μ	σ	$\frac{\sigma }{\left|\mu \right|}$
$K_{q_{x}/z_{m_{x}}}$	−0.0232	0.0127	0.5488	$8.8141\times 10^{-4}$	$7.9180\times 10^{-4}$	0.8983
$K_{q_{x}/z_{m_{y}}}$	−0.5474	0.0850	0.1553	$1.7863\times 10^{-4}$	0.0010	5.6959
$K_{q_{x}/z_{m_{z}}}$	−0.0911	0.0118	0.1296	$6.3377\times 10^{-4}$	$5.4550\times 10^{-4}$	0.8607
$K_{v_{z}/z_{m_{x}}}$	−0.0892	0.0157	1.6892	$4.5189\times 10^{-4}$	0.0020	4.4642
$K_{v_{z}/z_{m_{y}}}$	−0.0207	0.1250	6.0382	$-4.3975\times 10^{-4}$	0.0029	6.5964
$K_{v_{z}/z_{m_{z}}}$	0.0550	0.0931	1.6920	$-2.6585\times 10^{-4}$	0.0014	5.2806
$K_{b_{\omega_{y}}/z_{m_{x}}}$	−0.5344	0.0508	0.0950	$2.0447\times 10^{-6}$	$5.3013\times 10^{-7}$	0.2593
$K_{b_{\omega_{y}}/z_{m_{y}}}$	−0.0157	0.0413	2.6255	$-2.2956\times 10^{-7}$	$4.2771\times 10^{-7}$	1.8632
$K_{b_{\omega_{y}}/z_{m_{z}}}$	0.3143	0.0290	0.0923	$-3.4652\times 10^{-7}$	$1.3560\times 10^{-6}$	3.9131
